# Gender, Personality Traits and Experience With Psychiatric Patients as Predictors of Stigma in Italian Psychology Students

**DOI:** 10.3389/fpubh.2018.00362

**Published:** 2018-12-18

**Authors:** Leonardo Zaninotto, Jia Qian, Yao Sun, Giulia Bassi, Marco Solmi, Silvia Salcuni

**Affiliations:** ^1^Department of Mental Health, Local Health Unit n. 6 (“Euganea”), Padova, Italy; ^2^Department of Information Engineering, University of Padova, Padova, Italy; ^3^Department of Applied Mathematics and Computer Science, Technical University of Denmark, Kongens Lyngby, Denmark; ^4^Department of Developmental Psychology and Socialisation, University of Padova, Padova, Italy; ^5^Department of Neurosciences, University of Padova, Padova, Italy

**Keywords:** stigma, machine learning, psychology, student, personality

## Abstract

A sample of undergraduate Psychology students (*n* = 1005), prevalently females (82.4%), mean age 20.5 (sd 2.5), was examined regarding their attitudes toward people suffering from mental illness. The survey instrument included a brief form for demographic variables, the Attribution Questionnaire-9 (AQ-9), the Ten Items Personality Inventory (TIPI), and two questions exploring attitudes toward open-door and restraint-free policies in Psychiatry. Higher levels of stigmatizing attitudes were found in males (Pity, Blame, Help, and Avoidance) and in those (76.5%) who had never had any experience with psychiatric patients (Danger, Fear, Blame, Segregation, Help, Avoidance and Coercion). A similar trend was also found in those who don't share the policy of no seclusion/restraint, while subjects who are favorable to open-door policies reported higher Coercion scores. No correlations were found between dimensions of stigma and personality traits. A machine learning approach was then used to explore the role of demographic, academic and personality variables as predictors of stigmatizing attitudes. Agreeableness and Extraversion emerged as the most relevant predictors for blaming attitudes, while Emotional Stability and Openness appeared to be the most effective contributors to Anger. Our results confirmed that a training experience in Psychiatry might successfully reduce stigma in Psychology students. Further research, with increased generalizability of samples and more reliable instruments, should address the role of personality traits and gender on attitudes toward people suffering from mental illness.

## Introduction

The Canadian sociologist Erving Goffman defined stigma as “the situation of the individual who is disqualified from full social acceptance,” and characterized it as a relationship between “an attribute and a stereotype” [(1), p. 9]. In other words, stigma can be defined as a “mark” (attribute) that links a person to undesirable characteristics (stereotypes) (2) producing separation, status loss, and discrimination (3).

There are two main types of stigma against people suffering from mental illness: public stigma and self-stigma (4). Public stigma refers to the attitudes and beliefs held by the general public, while self-stigma occurs when the subjects endorse the negative public attitudes assigned to them (5). As a consequence of public stigma, people with mental illness suffer from discrimination in many areas of daily life (6, 7), while self-stigma may lead to reluctance to use mental health services (8–10).

Stigmatizing attitudes can also be found among mental health professionals (11–15), leading to poorer consumer's satisfaction (16) and poorer outcomes (17). Another possible source of stigma and a potential barrier against help seeking may be the negative image of Psychiatry deriving from the controversial issues of compulsory treatments and coercive practices. The semantic domain of seclusion and coercion is symbolically represented by the policy of locked doors in acute psychiatric wards, which further potentiates the notion of psychiatric patients as dangerous (18).

Among mental health professionals, psychologists are those who most directly get involved in relationship with the consumer, being also free from the charge of medications and compulsory treatments. Further, since in many contexts patients and caregivers tend to refer to less stigmatizing professionals first (19), psychologists are often regarded as the “front men” of mental health practitioners. Some studies showed that psychologists and psychiatrists might have more negative ratings than the general public on stereotypes, restriction of the individual's rights, and social distance (20, 21). Conversely, when compared to other mental health professionals, psychologists seem to have the lowest scores in negative emotions (anger, perceived dangerousness and fear) and in negative behavioral responses (coercion, segregation, and avoidance) (22).

Exploring possible predictors of stigmatizing attitudes among future professionals, such as Medicine or Psychology students, may be of crucial importance in order to define possible targets for anti-stigma interventions, as their attitudes and beliefs are supposed to be more easily modifiable (23). A growing body of evidence has shown that medical students usually express distancing attitudes toward people with mental illness (23–25), while Psychology students tend to define subjects with serious mental illness as unpredictable, antisocial and dangerous (26).

The primary aim of our study was to adopt Corrigan's (27) attributional model of public discrimination to explore the way undergraduate Psychology students perceive subjects with serious mental illness. Further, since a previous work by our group (28) has evidenced a relationship among professional variables, personality traits and avoidant attitudes toward patients in a sample of mental health professionals, our secondary aim was to apply a similar prediction model to a sample of Psychology students in order to detect possible associations among stigmatizing attitudes and: (a) some demographic and academic variables, and (b) some personality traits.

## Materials and Methods

### Subjects

The Inter-departments Research Ethics Committee of Psychology of the University of Padova approved our research protocol (nr. 2538/2018). The study was questionnaire-based and cross-sectional. The survey was conducted over two academic semesters (fall and spring) during the year 2017–2018, on a sample of undergraduate Psychology students from the University of Padova^1^. At Padova Psychology School there are four different undergraduate programmes: L1, Cognitive Psychology and Psychobiology; L2, Developmental and Educational Psychology; L3, Social and Work Psychology; L4, Psychology of Personality and Interpersonal Relationships. Study participants were enrolled from ten different classes across the three academic years: three classes from the 1^st^ year (L1, L3, L4), three classes from the 2nd year (L1, L3, L4), and four classes from the 3rd year (L1, L2, L3, L4). Two undergraduate students from the L4 program were employed to distribute the questionnaire to each class at the end of a lesson. Classes and lessons were chosen based on previous agreements between the professor and one the authors (SS). Approximately 1060 questionnaires were distributed; of these, 53 (5%) were returned back blank. Data collection was completely anonymous: no personal records about participants were collected, and no information about those who refused to take part in the study was gathered.

The recruitment procedure finally resulted in 1005 participants, prevalently females (82.39%), mean age 20.51 (*SD* = 2.50; range 18–47); all participants were unmarried. A description of the sample is reported in Table 1.

**Table 1:** Description of the sample, including demographic and academic features, personality traits, AQ-9 domains, and response to the Opinion Questions (OQ).

***n* = 1005**	**N/Mean**	**%/SD**
Females	828	82.39
Age	20.51	2.50
Education (years)	14.72	1.42
**Academic year**		
1st year	421	41.89
2nd year	269	26.77
3rd year	315	31.34
**Undergraduate programmes**		
L1	297	29.55
L2	99	9.85
L3	325	32.34
L4	284	28.26
**Previous experience in Psychiatry (*****n*** **=** **1003)**		
No	767	76.47
Yes	236	23.53
**I-TIPI**		
Extraversion (*n* = 999)	3.95	1.48
Agreeableness (*n* = 1000)	5.17	1.08
Coscientiousness (n = 995)	4.98	1.18
Emotional Stability (*n* = 996)	3.91	1.33
Openness to new experiences (*n* = 999)	4.84	1.15
**AQ-9 domains**		
Pity	5.85	1.87
Danger (*n* = 1003)	4.23	1.70
Fear (*n* = 999)	3.96	1.83
Blame (*n* = 997)	1.43	0.89
Segregation (*n* = 1003)	2.66	1.75
Anger (*n* = 1003)	1.41	0.93
Help (*n* = 996)	3.26	2.00
Avoidance (*n* = 995)	2.97	1.76
Coercion (*n* = 1004)	5.64	2.26
**OQ1 (*****n*** **=** **987)**		
Yes	884	89.56
No	103	10.44
**OQ2 (*****n*** **=** **989)**		
Yes	547	55.31
No	442	44.69

OQ1, Opinion Question 1: “Do you think in principle it would be possible to unlock the doors of acute psychiatric wards?”

OQ2, Opinion Question 2: “Do you think in principle it would be possibile to drop practices of seclusion and/or physical restraint in acute psychiatric wards?”

### Measures

The survey instrument included: a brief demographic form, a short version of the Attribution Questionnaire-27, the Attribution Questionnaire-9 (AQ-9) (29), two dichotomous (i.e., *yes/no*) Opinion Questions (OQ) exploring attitudes toward open-door and restraint-free policies in Psychiatry (OQ1: *Do you think in principle it would be possible to unlock the doors of acute psychiatric wards?* OQ2: *Do you think in principle it would be possible to give up on practices of seclusion and physical restraint in acute psychiatric wards?*), and the Ten Item Personality Inventory (TIPI) (30). All instruments were selected because of their simplicity and brevity, since a large number of items was supposed to increase respondent fatigue, measurement error, and misclassification. At the end of the booklet, a demographic form included the following items: age, gender, civil status, years of education, undergraduate program, academic year, and a question about any previous experience with psychiatric patients (i.e., stages in mental health services).

The AQ-9 (29) was developed after the AQ-27 (27, 31). The AQ-27 has been developed by Corrigan based on the Attribution Theory (32), and has been widely used in stigma research (33–37). It provides a clinical vignette describing an individual with schizophrenia (Harry) and asks the respondents to endorse their attitudes and beliefs toward Harry on a nine-point ordinal scale (9 = very much), with higher scores representing more stigmatizing attitudes. An Italian version of the AQ-27 has recently been validated (38). The AQ-9 was derived from the AQ-27 by extracting the nine items with the highest factor loadings, and it refers to the same domains as the AQ-27 (1 item = 1 domain): Pity (“I would feel pity for Harry”), Dangerousness (“How dangerous would you feel Harry is?”), Fear (“How scared of Harry would you feel?”), Blame (“I would think that it was Harry's own fault that he is in the present condition”), Segregation (“I think it would be best for Harry's community if he were put away in a psychiatric hospital”), Anger (“How angry would you feel at Harry?”), Help (“How likely is it that you would help Harry?”), Avoidance (“I would try to stay away from Harry”), and Coercion (“How much do you agree that Harry should be forced into treatment with his doctor even if he does not want to?”) (27, 31). No items are reverse scored, but for the “Help” item responses range from “definitely would help” (score = 1) to “definitely would not help” (score = 9). In our sample the Cronbach's alpha for the AQ-9 was 0.71.

Personality traits were evaluated using an Italian version (39) of the TIPI (30), a short instrument based on the Five-Factor Model (FFM) of personality (40), designed to assess the personality dimensions of Extraversion, Agreeableness, Conscientiousness, Emotional Stability and Openness to new experiences. The questionnaire consists of 10 items with a common stem “I see myself as” including two descriptors representing a pole of the Big-Five personality dimensions, for example: “*I see myself as dependable, self-disciplined*” (Item 3), “*I see myself as open to new experience, complex*” (Item 5). Each item is rated on a 7-point scale ranging from 1 (disagree strongly) to 7 (agree strongly). The score on each of the TIPI personality dimensions' subscales is measured, and ranges from 2 to 14. Although somewhat inferior to the standard Big-Five instruments, the TIPI takes about only 1 min to complete, and its convergent and discriminant validity, test–retest reliability, as well as patterns of external correlates has reached an adequate level (30).

### Statistical and Machine Learning Analysis

STATISTICA 6.0 software package (Dell Software, Tulsa, OK, USA) was used for descriptive statistics and linear correlations. All tests were two-tailed and significance was set with an alpha value of 0.05. Our main outcome variables (AQ-9 items) were processed by a series of Student's *t*-tests and one-way analysis of variance (ANOVAs) tests to detect possible differences across demographic and academic variables. Pearson product-moment correlation tests were also used to detect possible correlations with continuous variables, including TIPI personality dimensions. For the present study, only “moderate” to “strong” (*r* > 0.40) correlations were considered.

In recent years, machine learning approaches have gained interest in mental health as a method for building models to improve the diagnostic and therapeutic process (41, 42), to predict suicidality (43), as well as to analyse patterns of public stigma (44). Machine learning methods and, specifically, Gradient Boosting algorithms have been widely used in prediction models, to make decisions or to generate strategies (45–48), especially when there's no theory-driven framework about the potential relationships among variables (49).

To detect the most critical predictors for our outcome variables, we applied a Gradient Boosting Regressor (GBR) algorithm to our sample. GBR is a supervised machine learning algorithm based on a decision tree model. Decision trees are statistical models that recursively partition the input space in order to find rules, which are predictive of the output. The learning procedure consecutively fits new models to provide a more accurate estimate of the response variable.

In our GBR models target variables were all AQ-9 items, while input variables were gender, academic year, undergraduate course and personality traits. Python 3.0 software package (Python Software Foundation, Wilmington, DE, USA) was used for machine learning.

## Results

A description of the sample is reported in Table 1. The majority of subjects was recruited among 1st year students. The L1 program included a higher proportion of males compared to the others (Chi-sq = 16.11, d.f. = 3, *p* = 0.001), while no significant difference was found in the male/female ratio across academic years. More than three in four had never had any experience with psychiatric patients, and gender or choice of undergraduate program had no effect on this ratio. Conversely, almost one in three students attending the 3rd year had already had at least one experience in Psychiatry (1st year = 21.72% vs. 2nd year = 18.22% vs 3rd year = 30.48%; Chi-sq = 13.43, d.f. = 2, *p* = 0.001). Female students were younger (20.43 ± 2.45 vs. 20.88 ± 2.69; *t* = −2.19, d.f. = 1003, *p* = 0.029) and reported higher scores on the personality traits of Agreeableness (5.23 ± 1.09 vs. 4.93 ± 1.00, *t* = 3.30, d.f. = 998, *p* = 0.001) and Conscientiousness (5.04 ± 1.18 vs. 4.71 ± 1.14, *t* = 3.42, d.f. = 993, *p* = 0.001), while male students reported higher scores on Emotional Stability (4.31 ± 1.41 vs. 3.82 ± 1.30, *t* = −4.48, d.f. = 994, *p* < 0.001).

Regarding opinion questions (Table 1), the majority of survey respondents (89.56%) declared to be in favor of unlocking the doors of acute psychiatric wards (OQ1). A higher proportion of favorable subjects was found among students attending the 1st year (1st year = 93.46% vs. 2nd year = 90.19% vs. 3rd year = 83.82%, Chi-sq = 17.74, d.f. = 2, *p* < 0.001) and the L1 class (L1 = 94.16% vs. L2 = 88.89% vs. L3 = 85.27% vs. L4 = 89.93%, Chi-sq = 12.96, d.f. = 3, *p* = 0.005). Conversely, opinions about the practice of restraint (OQ2) were not affected by academic year or undergraduate program. A history of previous direct experience with psychiatric patients resulted in no significant effect on answers to either OQ1 or OQ2.

As regards personality traits, a small significant difference was found across years in terms of Openness, with the highest levels in the 1st year (1st year = 4.96 ± 1.18 vs. 2nd year = 4.80 ± 1.08 vs 3rd year = 4.72 ± 1.14; *F* = 4.21, d.f. = 2.996, *p* = 0.015). Some differences across undergraduate programmes were also found in terms of Openness (L1 = 4.97 ± 1.13 vs. L2 = 4.61 ± 1.05 vs. L3 = 4.77 ± 1.18 vs. L4 = 4.86 ± 1.14; *F* = 3.01, d.f. = 3.995, *p* = 0.029) and Agreeableness (L1 = 5.14 ± 1.09 vs. L2 = 5.20 ± 1.17 vs. L3 = 5.05 ± 1.05 vs. L4 = 5.34 ± 1.06; *F* = 3.79, d.f. = 3.996, *p* = 0.010), while a history of previous experiences in Psychiatry was associated to higher levels of Extraversion (4.17 ± 1.42 vs. 3.88 ± 1.49, *t* = 2.63, d.f. = 995, *p* = 0.009). Higher scores on Openness were found in those who declared to be in favor of open-doors (4.87 ± 1.12 vs. 4.57 ± 1.33, *t* = 2.51, d.f. = 979, *p* = 0.012) and no-restraint policies (4.93 ± 1.10 vs. 4.74 ± 1.18, *t* = 2.62, d.f. = 981, *p* = 0.009), the latest also reporting higher scores on Conscientiousness (5.14 ± 1.14 vs. 4.86 ± 1.19, *t* = −3.72, d.f. = 978, *p* < 0.001).

Exploring the effect of demographic and academic variables on AQ-9 domains (Table 2), we found that male students scored significantly higher on Pity, Blame, Help and Avoidance, while no relevant effect was found for age or duration of education. Students attending the L1 program showed higher scores on Pity and lower scores on Danger, while the L2 program was associated to higher Coercion scores. Those who answered positively to the OQ1 resulted to be higher in Coercion, while those who declared to be in favor of no-restraint policies (OQ2) were lower in all stigmatizing attitudes except Pity and Anger. Finally, a previous experience with psychiatric patients was associated to lower scores on Danger, Fear, Segregation, Help, and Avoidance.

**Table 2:** Effect of gender and academic variables on AQ-9 domains.

	**Pity**	**Danger**	**Fear**	**Blame**	**Segregation**	**Anger**	**Help**	**Avoidance**	**Coercion**
**GENDER**									
Females	5.76 (1.89)	4.23 (1.69)	4.01 (1.84)	1.40 (0.86)	2.68 (1.79)	1.40 (0.92)	3.17 (2.00)	2.91 (1.73)	5.69 (2.26)
Males	6.28 (1.71)	4.24 (1.72)	3.74 (1.75)	1.60 (1.01)	2.58 (1.54)	1.46 (0.97)	3.70 (1.93)	3.25 (1.85)	5.40 (2.26)
*t (p)*	−3.41 **(0.001)**	−0.05 (n.s.)	1.74 (n.s.)	−2.7 **(0.007)**	0.71 (n.s.)	−0.86 (n.s.)	−3.21 **(0.001)**	−2.34 **(0.019)**	1.53 (n.s.)
**ACADEMIC YEAR**									
1st year	5.75 (1.90)	4.15 (1.73)	3.85 (1.88)	1.46 (0.95)	2.70 (1.77)	1.37 (0.98)	3.31 (2.01)	3.03 (1.83)	5.55 (2.34)
2nd year	6.10 (1.75)	4.38 (1.71)	4.14 (1.78)	1.52 (0.83)	2.74 (1.80)	1.46 (0.94)	3.29 (1.98)	2.96 (1.71)	5.89 (2.15)
3rd year	5.76 (1.91)	4.21 (1.63)	3.95 (1.79)	1.42 (0.86)	2.55 (1.69)	1.41 (0.85)	3.16 (2.00)	2.89 (1.71)	5.53 (2.22)
*F (p)*	3.41 **(0.033)**	1.47 (n.s.)	2.09 (n.s.)	0.21 (n.s.)	1.00 (n.s.)	0.71 (n.s.)	0.53 (n.s.)	0.57 (n.s.)	2.35 (n.s.)
**UNDERGRADUATE PROGRAMMES**									
L1	6.06 (1.80)	4.00 (1.55)	3.89 (1.83)	1.45 (0.86)	2.49 (1.59)	1.33 (0.80)	3.30 (2.02)	2.98 (1.82)	5.73 (2.18)
L2	5.54 (1.82)	4.18 (1.53)	3.91 (1.71)	1.39 (0.91)	2.69 (1.73)	1.34 (0.67)	3.02 (1.87)	2.68 (1.52)	6.12 (2.05)
L3	5.69 (1.90)	4.36 (1.83)	4.05 (1.90)	1.48 (0.93)	2.81 (1.86)	1.48 (1.05)	3.33 (2.02)	3.07 (1.77)	5.42 (2.43)
L4	5.90 (1.90)	4.35 (1.72)	3.95 (1.80)	1.38 (0.89)	2.66 (1.78)	1.43 (0.98)	3.22 (1.99)	2.94 (1.75)	5.62 (2.19)
*F (p)*	3.41 **(0.017)**	2.98 **(0.031)**	0.41 (n.s.)	0.75 (n.s.)	1.75 (n.s.)	1.64 (n.s.)	0.69 (n.s.)	1.28 (n.s.)	2.65 **(0.048)**
**PREVIOUS EXPERIENCE**									
Yes	5.75 (1.93)	3.99 (1.74)	3.56 (1.78)	1.44 (0.93)	2.37 (1.70)	1.39 (1.02)	2.97 (1.91)	2.54 (1.50)	5.42 (2.24)
No	5.88 (1.85)	4.31 (1.68)	4.09 (1.83)	1.43 (0.88)	2.75 (1.76)	1.41 (0.90)	3.35 (2.02)	3.10 (1.81)	5.71 (2.26)
*t (p)*	−0.91 (n.s.)	−2.57 **(0.010)**	−3.83 **(< 0.001)**	0.1 (n.s.)	−2.93 **(0.003)**	−0.34 (n.s.)	−2.58 **(0.010)**	−4.29 **(< 0.001)**	−1.72 (n.s.)
**OQ1**									
Yes	5.84 (1.87)	4.22 (1.70)	3.96 (1.82)	1.42 (0.88)	2.67 (1.73)	1.39 (0.91)	3.26 (2.01)	2.93 (1.73)	5.71 (2.22)
No	5.78 (1.91)	4.34 (1.66)	3.86 (1.94)	1.52 (0.97)	2.64 (1.92)	1.46 (1.01)	3.33 (2.01)	3.24 (1.94)	5.14 (2.41)
*t (p)*	0.34 (n.s.)	−0.68 (n.s.)	0.49 (n.s.)	−1.11 (n.s.)	0.14 (n.s.)	−0.66 (n.s.)	−0.33 (n.s.)	−1.68 (n.s.)	2.43 **(0.015)**
**OQ2**									
Yes	5.78 (1.88)	4.06 (1.70)	3.78 (1.81)	1.38 (0.81)	2.40 (1.67)	1.39 (0.98)	3.08 (1-96)	2.76 (1.63)	5.40 (2.27)
No	5.95 (1.86)	4.40 (1.66)	4.19 (1.81)	1.50 (1.00)	2.99 (1.80)	1.43 (0.86)	3.45 (2.02)	3.18 (1.85)	5.95 (2.20)
*t (p)*	−1.49 (n.s.)	−3.48 **(0.001)**	−3.54 **(< 0.001)**	−2.18 **(0.030)**	−5.31 **(< 0.001)**	−0.68 (n.s.)	−2.85 **(0.005)**	−3.80 **(< 0.001)**	−3.85 **(< 0.001)**

Statistics for each AQ-9 item are reported under means and SDs (in brackets).

*Significant p-values are reported in bold*.

As regards bivariate correlations (Table 3), perceived dangerousness (Danger) showed a significant positive correlation with negative emotions (Fear) and negative behavioral responses (Segregation, Avoidance and Coercion). Fear was also positively correlated with Segregation and Avoidance, while avoidant attitudes increased together with Segregation and Help. Although some significant correlations were found between some personality traits and the AQ-9 domains, they were in the range of “very weak” (< 0.19) linear relationships.

**Table 3:** Bivariate correlations expressed by *r* and *p* values (in brackets) among AQ-9 items and personality traits.

***n* = 940 casewise deletion of missing data**	**Pity**	**Danger**	**Fear**	**Blame**	**Segregation**	**Anger**	**Help**	**Avoidance**	**Coercion**	**Extraversion**	**Agreeable-ness**	**Coscientious-ness**	**Emotional Stability**	**Openness**
Pity	1.00 (*p* = —)													
Danger	0.26 (*p* < 0.001)	1.00 (*p* = —)												
Fear	0.21 (*p* < 0.001)	**0.63** ** (*****p*** **=** **0.00)**	1.00 (*p* = —)											
Blame	0.01 p = n.s.	0.13 (*p* < 0.001)	0.11 (*p* < 0.001)	1.00 (*p* = —)										
Segregation	0.08 (*p* = 0.01)	**0.37** **(*****p*** **=** **0.00)**	**0.38** **(*****p*** **=** **0.00)**	0.24 (*p* < 0.001)	1.00 (*p* = —)									
Anger	0.07 (*p* = 0.02)	0.20 (*p* < 0.001)	0.20 (*p* < 0.001)	0.25 (*p* < 0.001)	0.27 (*p* < 0.001)	1.00 (*p* = —)								
Help	0.03 (*p* = 0.33)	0.15 (*p* < 0.001)	0.23 (*p* < 0.001)	0.12 (*p* < 0.001)	0.18 (*p* < 0.001)	0.08 (*p* = 0.01)	1.00 (*p* = —)							
Avoidance	0.11 (*p* = 0.001)	**0.41** **(*****p*** **=** **0.00)**	**0.48** ** (*****p*** **=** **0.00)**	0.21 (*p* < 0.001)	**0.31** **(*****p*** **=** **0.00)**	0.28 (*p* < 0.001)	**0.44** ** (*****p*** **=** **0.00)**	1.00 (*p* = —)						
Coercion	0.11 (*p* = 0.001)	**0.30** **(*****p*** **=** **0.00)**	0.28 (*p* < 0.001)	0.01 *p* = n.s.	0.22 (*p* < 0.001)	0.10 (*p* = 0.002)	0.13 (*p* < 0.001)	0.21 (*p* < 0.001)	1.00 (*p* = —)					
Extraversion	0.02 (*p* = n.s.)	0.03 (*p* = n.s.)	0.00 (*p* = n.s.)	0.06 (*p* = n.s.)	0.03 (*p* = n.s.)	0.03 (*p* = n.s.)	0.00 (*p* = n.s.)	−0.06 (*p* = n.s.)	0.09 (*p* = 0.004)	1.00 (*p* = —)				
Agreeableness	0.07 (*p* = 0.039)	0.03 (*p* = n.s.)	0.04 (*p* = n.s.)	0.00 (*p* = n.s.)	−0.03 (*p* = n.s.)	−0.06 (*p* = 0.049)	−0.01 (*p* = n.s.)	−0.07 (*p* = 0.046)	0.09 (*p* = 0.007)	−0.09 (*p* = 0.004)	1.00 (*p* = —)			
Conscientiousness	−0.04 (*p* = n.s.)	0.03 (*p* = n.s.)	0.06 (*p* = n.s.)	−0.02 (*p* = n.s.)	0.05 (*p* = n.s.)	−0.08 (*p* = 0.016)	−0.03 (*p* = n.s.)	−0.06 (*p* = n.s.)	0.14 (*p* < 0.001)	−0.11 (*p* = 0.001)	0.18 (*p* < 0.001)	1.00 (*p* = —)		
Emotional Stability	−0.06 (*p* = n.s.)	−0.01 (*p* = n.s.)	−0.04 (*p* = n.s.)	0.03 (*p* = n.s.)	0.05 (*p* = n.s.)	−0.04 (*p* = n.s.)	0.08 (*p* = 0.016)	−0.01 (*p* = n.s.)	−0.04 (*p* = n.s.)	0.03 (*p* = n.s.)	0.17 (*p* < 0.001)	0.15 (*p* < 0.001)	1.00 (*p* = —)	
Openness	−0.01 (*p* = n.s.)	−0.10 (*p* = 0.002)	−0.16 (*p* < 0.001)	−0.01 (*p* = n.s.)	−0.16 (*p* < 0.001)	−0.06 (*p* = 0.048)	−0.15 (*p* < 0.001)	−0.19 (*p* < 0.001)	−0.09 (*p* = 0.009)	0.26 (*p* < 0.001)	0.12 (*p* < 0.001)	−0.10 (*p* = 0.003)	0.03 (*p* = n.s.)	1.00 (*p* = —)

*Significant moderate-to-high correlations are evidenced in bold*.

Hence, we introduced GBR as a complementary approach to explore the latent relationship among all the aforementioned variables. Given the fact that OQ items were well-explained by their association with AQ-9 dimensions, they were not included in our models.

**Figure 1:**
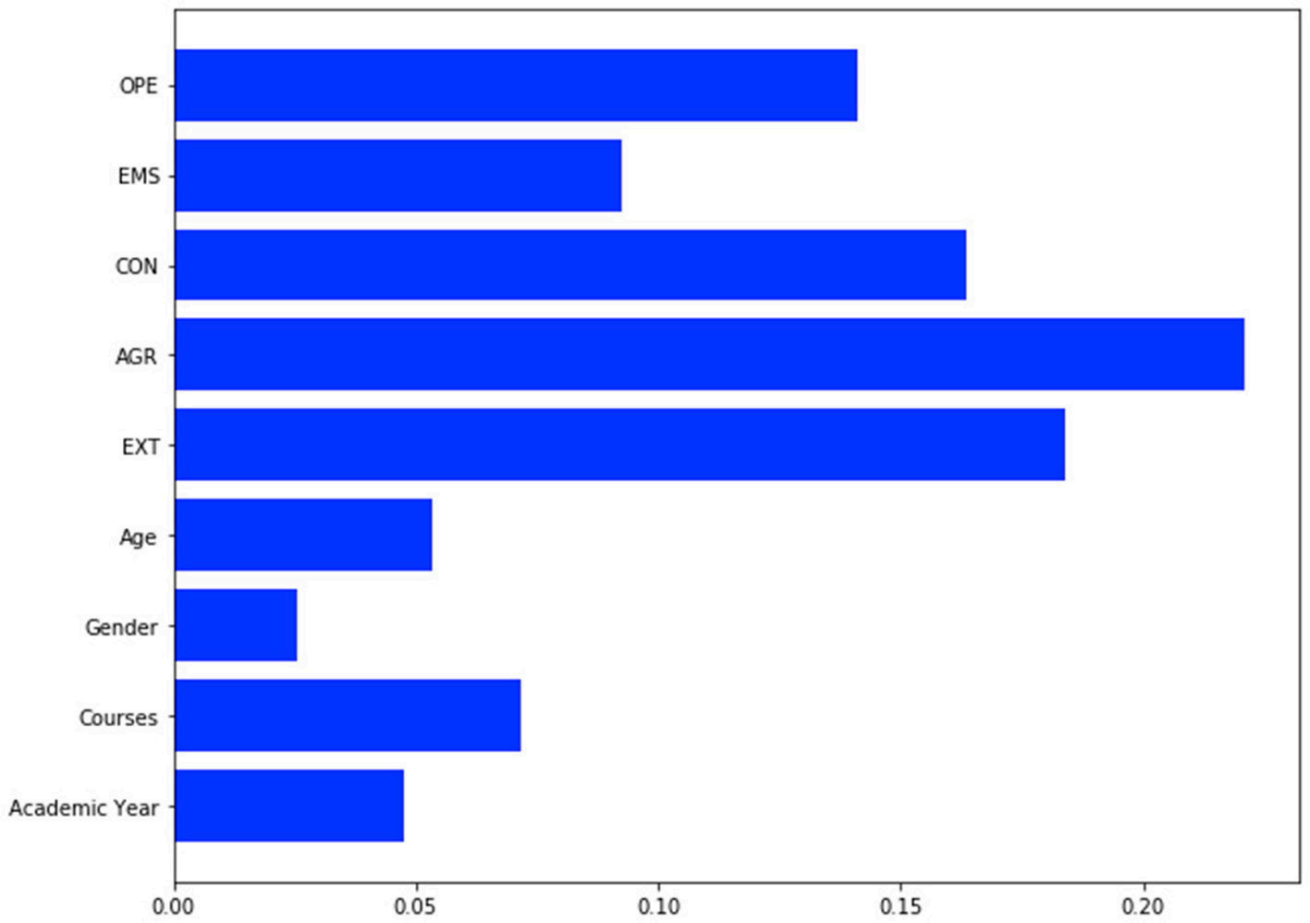
GBR prediction model for Blame item. OPE, Openness; EMS, Emotional Stability; CON, Conscientiousness; AGR, Agreeableness; EXT, Extraversion. Accuracy for Blame is 0.656.

**Figure 2:**
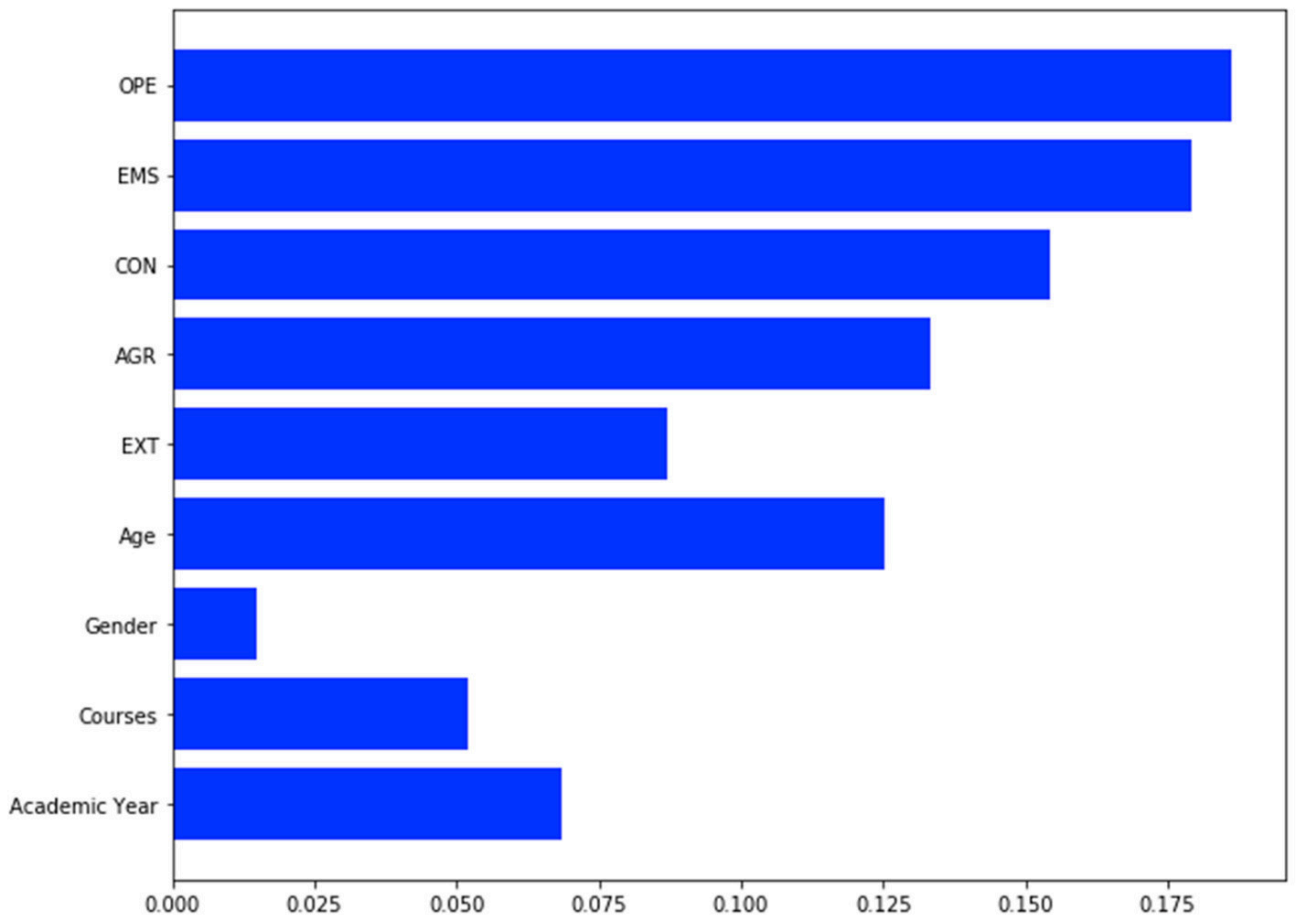
GBR prediction model for Anger item. OPE, Openness; EMS, Emotional Stability; CON, Conscientiousness; AGR, Agreeableness; EXT, Extraversion. Accuracy for Anger is 0.709.

By leveraging the Machine Learning technique, Blame and Anger resulted to be the most predictable targets, their accuracy being 65.6 and 70.9%, respectively. According to our models, Agreeableness and Extraversion emerged as the most relevant predictors for Blame (Figure 1), while Emotional Stability and Openness to new experiences emerged as the most effective contributors to Anger (Figure 2).

## Discussion

This study explores the role of some demographic, academic and personality features on the development of stigmatizing attitudes in a large sample of undergraduate Psychology students. Machine learning was adopted as a complementary tool to explore any association among variables that could not be detected by traditional statistical methods.

First glance, our findings seem to support the view of a “gender effect” on stigma (50–52), since male students reported significantly higher scores on several AQ-9 items (Pity, Blame, Help and Avoidance) when compared to their female peers. No association could be gathered for the other demographic variables (age, level of education and civil status), probably because of the homogeneity of the sample.

However, attitudes toward people with mental illness may vary to a small extent only depending on socio-demographic characteristics, and findings about a specific “gender effect” have shown to be quite inconsistent (53). Thus, a higher level of negative attitudes in male students may also depend on other factors, such as a different conceptualization of mental illness. Indeed, women are more likely to endorse psychosocial conceptualizations instead of biological explanations of mental illness (54), and when compared to other causal explanations, a biological understanding of mental health problems has been repeatedly associated to more negative attitudes toward patients (26, 55–58).

In our sample, a higher proportion of males was found in the L1 program, which is supposed to provide a cognitive and neurobiological approach to mental health problems, but the same class also included a higher proportion of subjects who declared to be in favor of open-door policies in Psychiatry (OQ1). Further, L1 students reported less perceived dangerousness (Danger), and more piteous attitude (Pity) than their peers, while those who enrolled in the L2 program scored higher in Coercion. Thus, these findings seem to suggest that biogenetic causal models of mental illness, when compared to other models (i.e., developmental and educational), may not only be associated to more negative attitudes in general, but they may as well reduce notions of self-responsibility and subsequently evoke less negative responses such as pity and help (59). Nevertheless, since no specific instrument was adopted to assess opinions about mental illness, we can only make a tentative but challenging hypothesis about a possible association among gender, choice of academic profile and attitudes toward people with mental illness.

Some personality traits, such as Openness and Agreeableness, resulted to play a major role on stigmatizing attitudes. Indeed, although bivariate correlations were in the range of very weak associations, according to our machine learning algorithms, Agreeableness and Extraversion were predictive of blaming attitudes (Blame), while Emotional Stability and Openness to new experiences emerged as the most effective contributors to Anger (the direction of effect deriving from GBR algorithms could not be determined, but may be partly inferred from significant bivariate correlations). Further, higher scores on Openness were found in those who declared to be in favor of open-doors and no-restraint policies in Psychiatry.

To our knowledge there are very few studies addressing the relationship between personality and mental health related stigma. A recent work by our group (28) on a sample of mental health professionals evidenced a negative correlation between Openness and avoidant attitudes toward patients. A previous study by Brown et al. (60) on a sample of college students, found that Openness predicted a decreased perception of dangerousness and less social distancing, while lower scores on Agreeableness predicted a negative emotionality toward subjects suffering from mental illness. Interestingly, Openness and Agreeableness were also found to be positively associated to empathy toward patients in a sample of medical students (61).

Finally, a previous experience with psychiatric patients (i.e., a training experience in a psychiatric unit) was associated to lower scores on Danger, Fear, Segregation, Help and Avoidance. Fear, perceived dangerousness and desire for social distance are supposed to decrease as familiarity with psychiatric patients increases (62). A recent review by Yamagughi et al. (63) has evidenced that the most effective interventions to reduce mental health related stigma in university and college students were those implying any kind of contact. A contact element, even of indirect nature (i.e., video- or audio-taped testimonies), may be the most relevant factor in tackling the stigma attached to mental illness (64–66).

The main limitation of our study is represented by its cross-sectional design. The generalizability of results cannot be assumed due to the limited representativeness of the sample, which prevalently comprised young females with a high level of education. Another limitation is the lack of information about those subjects who did not take part in the study, since students who did not choose to participate might have vastly different opinions on stigma. However, the sample's homogenous nature might have been important for our results on the role personality, because FFM traits are supposed to be characterized by unique changes during the emerging adulthood phase (67, 68). Additionally, our findings are based on self-reported attitudes, which inherently have risk of response bias, including social desirability. Familiarity with mental health problems was only explored through the indirect index of training experiences with psychiatric patients, while personal experience with mental illness (i.e., a family member) was not considered. Finally, unlike the traditional statistical approach, the relationship between predictors and variables in machine learning models are rather vague, and the interpretation and explanation of results generated by such processes may be challenging. However, as claimed Woo et al. (69), the use of innovative technique starts with testing and exploration.

Notwithstanding these limitations, this study provides evidence that: (a) male Psychology students may report greater negative attitudes toward patients than their female peers; (b) any direct experience with psychiatric patients may have a significant effect in lessening stigmatizing attitudes; (c) some personality traits, such as Agreeableness and Openness to new experiences may have a relevant role in the development of some components of mental health stigma.

These results seem to confirm that a training experience including a direct personal experience with psychiatric patients may exert a substantial influence on shaping less negative attitudes toward mental illnesses and Psychiatry. Our findings seem also to suggest that the personality of students should be taken into account in developing anti-stigma programs in undergraduate education. Further research, with increased generalizability of samples and more valid measures should be undertaken to disentangle the complex relationship among demographic features, academic variables, personality traits and attitudes toward people suffering from mental illness.

## Author Contributions

All the authors actively contributed to the production of the research paper. LZ, SS, and MS developed the research project. JQ, YS, and GB contributed to statistical analyses. All authors participated in writing the paper.

### Conflict of Interest Statement

The authors declare that the research was conducted in the absence of any commercial or financial relationships that could be construed as a potential conflict of interest.

## Footnotes

^1^The higher education system in Italy adopts a 3+2 organization, where first level (Laurea Triennale) may correspond to a Bachelor Degree, while the two additional years (Laurea Magistrale) may be assimilated to a Master Degree.
